# Alterations in the RTK/Ras/PI3K/AKT pathway serve as potential biomarkers for immunotherapy outcome of diffuse gliomas

**DOI:** 10.18632/aging.203102

**Published:** 2021-06-08

**Authors:** Song Han, Peng-Fei Wang, Hong-Qing Cai, Jing-Hai Wan, Shou-Wei Li, Ze-Huan Lin, Chun-Jiang Yu, Chang-Xiang Yan

**Affiliations:** 1 Department of Neurosurgery, Sanbo Brain Hospital, Capital Medical University, China; 2 Department of Neurosurgery, National Cancer Center/Cancer Hospital, Chinese Academy of Medical Sciences and Peking Union Medical College, China; 3 Grade 2018, Medical College, Qingdao University, Qingdao, China

**Keywords:** gliomas, immunotherapy, small-molecule kinase inhibitors, bioinformatic analysis

## Abstract

Background: Diffuse gliomas are the most common malignant brain tumors, and immune checkpoint inhibitors have limited therapeutic effects against this cancer. Three oncogenic pathways are altered in diffuse gliomas: the RTK/Ras/PI3K/AKT signaling, TP53, and RB pathways. Although these pathways may affect the tumor immune microenvironment, their association with immunotherapy biomarkers remains unclear.

Methods: We used copy number variation and mutation data to stratify patients with specific oncogenic signaling alterations, and evaluated their correlation with predictive immunotherapy biomarkers, including tumor mutation burden (TMB), immune cytolytic activity (CYT), tumor purity, and tumor-infiltrating CD8^+^ T cells. Immune checkpoint expression and interferon-γ signaling activity were also compared in these samples.

Results: We identified differentially expressed genes in three distinct oncogenic pathways. Gene ontology analysis of these genes revealed the involvement of RTK/Ras/PI3K/AKT-associated genes in immune and inflammatory responses. Moreover, significantly elevated TMB, CYT, and numbers of CD8^+^ T cells and decreased tumor purity were correlated with altered RTK/Ras/PI3K/AKT signaling. Single cell sequencing also confirmed that this tumor subgroup had increased immune checkpoint expression and interferon-γ signaling activity. Immune phenotyping based on the presence of CD274 and TMB or CD274 and CD8 T^+^ cells indicated that tumors with altered RTK/Ras/PI3K/AKT pathways represent a beneficial subtype and are associated with improved survival.

Conclusion: Altered RTK/Ras/PI3K/AKT signaling and immunotherapy biomarkers are strongly correlated in gliomas. Gliomas with altered expression of RTK/Ras/PI3K/AKT pathway components may be sensitive to immunotherapy. A combination of small-molecule kinase inhibitors and immunotherapy is proposed for this subgroup of tumors.

## INTRODUCTION

Glioblastoma (GBM) is the most prevalent and a deadly primary malignant central nervous system tumor [1]. Genomic profiling has identified three major pathways that are deregulated in GBM: the RTK/Ras/PI3K/AKT signaling, TP53, and RB pathways [2–5]. Many drugs that target these three signaling pathways. However, only a few patients respond to these drugs [6]. Therapeutic failure occurs because of intra-tumor heterogeneity and a lack of effective therapeutic strategies, such as combination therapies [6, 7].

The tumor microenvironment (TME), which involves interactions between immune cells and tumor cells, play pivotal roles in glioma progression [8]. The TME may be shaped by the oncogenic signaling pathways of tumor cells [8, 9]. PTEN deficiency and activation of phosphatidylinositol-3-OH kinase (PI3K) could aggravate CD274 expression and weaken the function of tumor-associated T cells in gliomas [10]. Loss of PTEN in glioblastoma cells increases macrophage infiltration, subsequently supporting glioma-cell survival and promoting angiogenesis [9]. TP53 mutations are associated with increased expression of immune checkpoint genes and activation of effector T cells in lung adenocarcinoma [11]. These findings indicate that combination of oncogenic signaling-pathway inhibitors and immunotherapy is a promising therapeutic strategy for gliomas. One preclinical model suggests that a combination therapy targeting TP53 and PD-1 can kill glioma cells more effectively than monotherapy against either of these targets [12]. Consequently, improving the understanding of the association between oncogenic signaling pathways and the TME in gliomas may lead to the development of therapeutic strategies for patients with gliomas.

This study was conducted to explore the potential changes in the TME caused by alterations in GBM oncogenic signaling pathways due to gene mutations, fusions, or copy number variations. The TME was described based on the expression of immune check point genes, immune-cell infiltration, tumor mutation burden (TMB), immune cytolytic activity (CYT), tumor purity, and tumor infiltrating CD8^+^ T cells; all of which are predictive biomarkers for immunotherapy.

## RESULTS

### Identification of RTK/Ras/PI3K/AKT signaling-related biological processes

**Figure 1:**
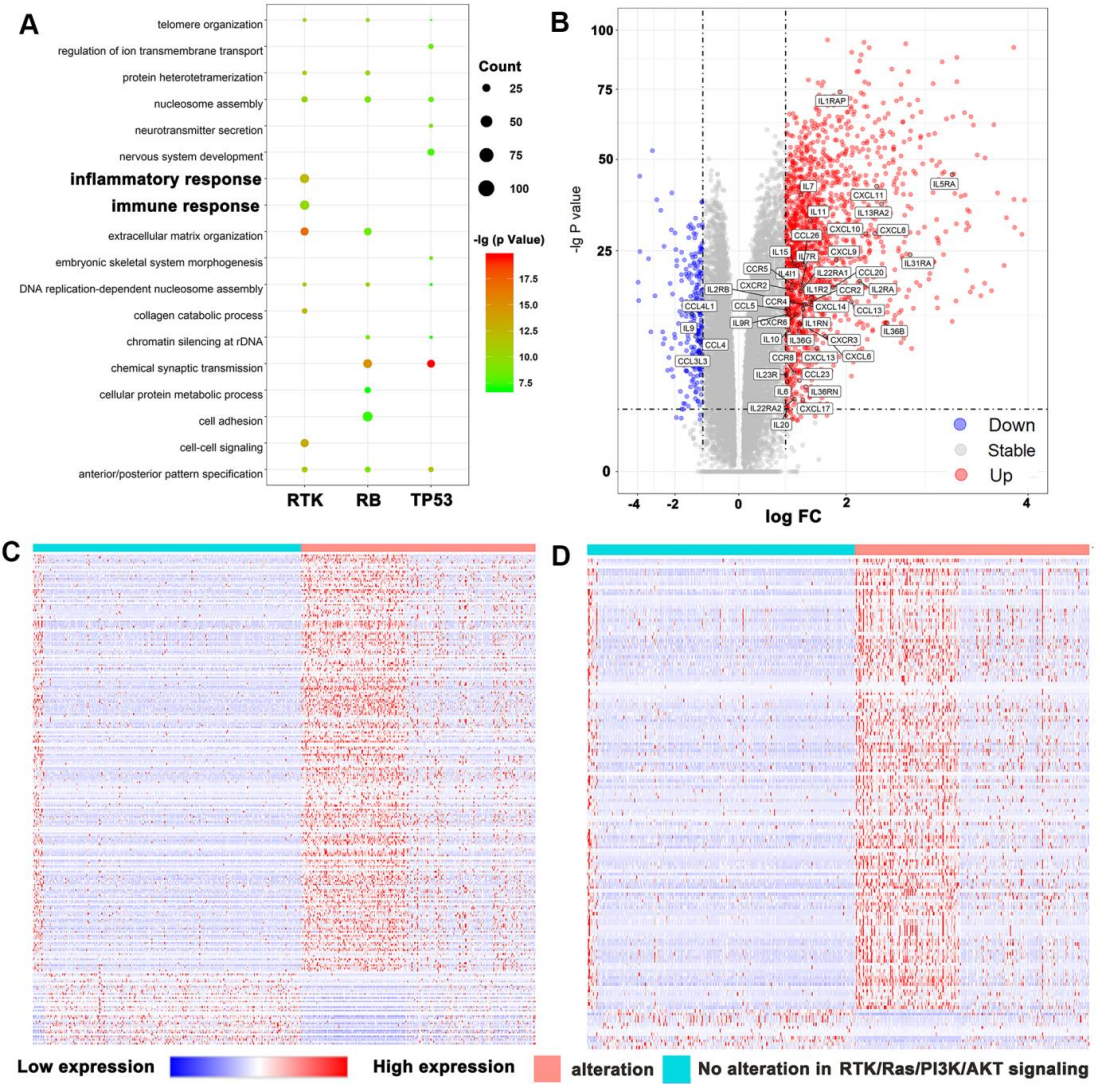
**RTK/Ras/PI3K/AKT pathway was strongly related to immune functions in glioma.** (**A**) GO analysis showed that RTK/Ras/PI3K/AKT pathway was involved in immune response and inflammatory response. (**B**) Volcano plot display differentially expressed cytokines and chemokines according to RTK/Ras/PI3K/AKT signaling. Heat maps display the association between RTK/Ras/PI3K/AKT pathway, and most correlated gene expression in the immune response (**C**) and inflammatory response (**D**).

We analyzed differential gene expression data from TCGA database and identified numerous DEGs associated with alterations in the RTK/Ras/PI3K/AKT signaling, RB, and TP53 pathways. The genes involved in these three oncogenic signaling pathways were described by cBioPortal and are listed in Supplementary Table 1. GO analysis was performed for the identified DEGs using DAVID and showed that alterations in RTK/Ras/PI3K/AKT signaling were involved in the inflammatory and immune responses (Figure 1A). Moreover, there were abundant cytokines and chemokines that were elevated in tumors with activated RTK/Ras/PI3K/AKT signaling (Figure 1B). Notably, the gene expression levels of interferon-stimulated chemokines (CXCL9/10/CXCL11), which recruit activated T and NK cells [13, 14], were significantly increased in tumors with altered RTK/Ras/PI3K/AKT signaling.

Next, we downloaded the genes that are related to the immune response (GO: 0006955) or the inflammatory response (GO: 0006954) from the AmiGO 2 website (http://amigo.geneontology.org/amigo). We matched the downloaded genes with DEGs according to the status of the RTK/Ras/PI3K/AKT pathway. We identified 228 upregulated and 40 downregulated genes involved in the immune response (Supplementary Table 2) and 135 upregulated and 12 downregulated genes involved in the inflammatory response (Supplementary Table 3). All the DEGs from the two biological process were selected for heatmap analysis (Figure 1C, D).

### Relationship between the RTK/Ras/PI3K/AKT signaling status and predictive biomarkers

**Figure 2:**
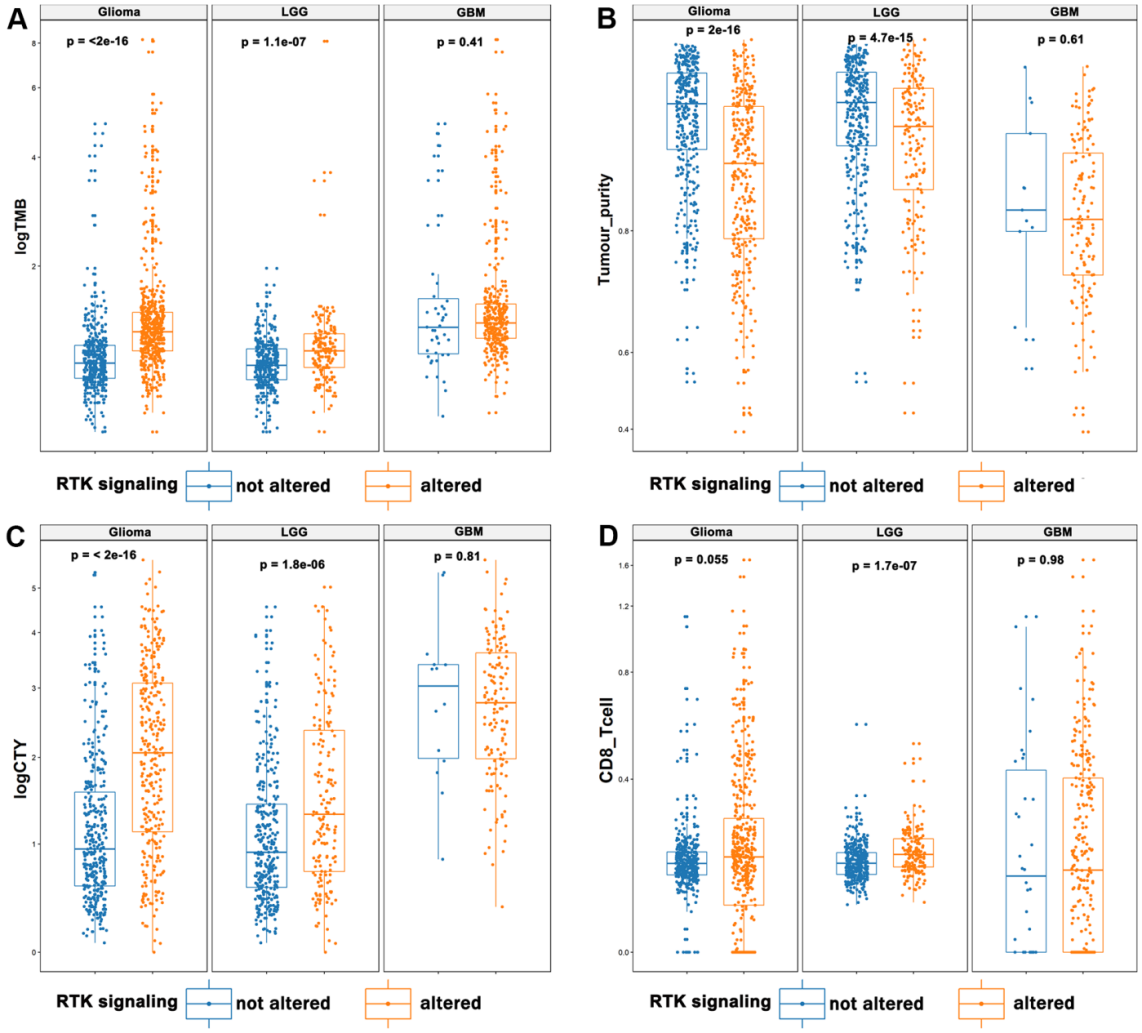
**The landscape of immunotherapy predictive biomarkers in association with RTK/Ras/PI3K/AKT pathway.** It displayed that elevated TMB (**A**), CYT (**C**), and infiltrating CD8+ T cells (**D**), and decreased tumor purity (**B**) in the activated RTK/Ras/PI3K/AKT pathway.

There were data indicating that lower tumor purity is associated with an intense local immune response [15]. Here, we found that TMB was significantly increased in diffuse gliomas and Lower grade gliomas (LGGs), in which the RKT signaling pathway was activated (*p* < 0.001, Figure 2A). Tumors with altered RTK also showed elevated levels in gliomas and LGG (*p* < 0.001, Figure 2B). CYT reflected the immune cytolytic activity in gliomas and was tremendously increased in cancer patients treated with immune checkpoint inhibitors (ICIs) [16, 17]. Our study also showed that CYT was significantly upregulated in tumors with altered RTK/Ras/PI3K/AKT signaling (*p* < 0.001, Figure 2C). Moreover, we showed that tumors with activated RTK/Ras/PI3K/AKT pathway were enriched with LGG-infiltrating CD8^+^ T cells (*p* <0.001, Figure 2D). However, we also found significant differences in immunotherapy biomarkers between LGG and GBM, the reason for which is unclear and requires further analysis. The tumor purity, TMB, CYT, and tumor infiltrating CD8^+^ T cells were all compared by the Wilcoxon test.

### Correlations between RTK/Ras/PI3K/AKT signaling and immune-regulatory gene-transcript signatures

**Figure 3:**
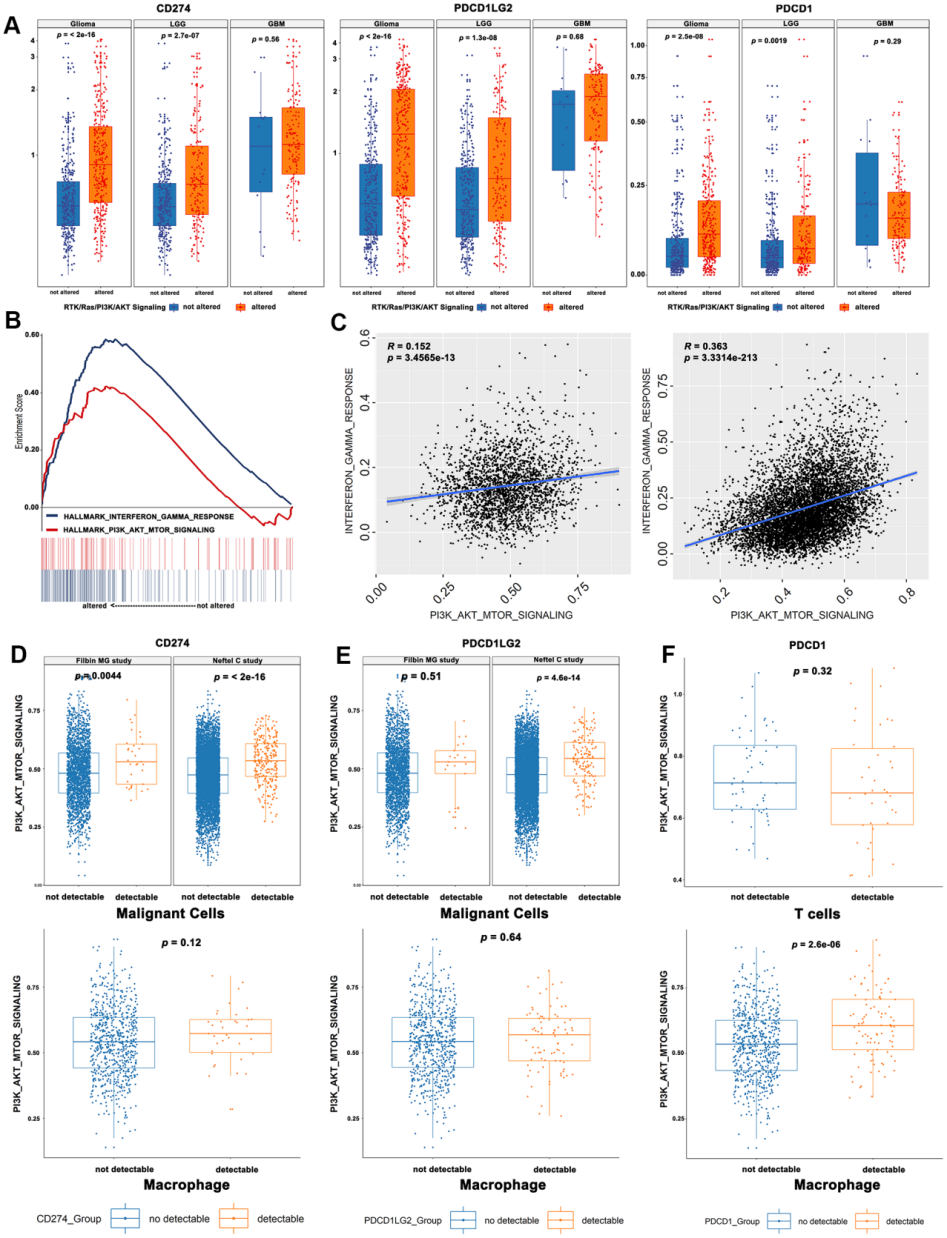
**Correlations between RTK/Ras/PI3K/AKT signaling and immune-regulatory genes mRNA signatures.** (**A**) The immune checkpoint genes were significantly upregulated in the activated RTK/Ras/PI3K/AKT signaling. (**B**) GSEA analysis showed a prominent enrichment of IFNγ pathways and PI3K-AKT-mTOR. (**C**) The single cell RNA seq showed a significant correlation between PI3K-AKT-mTOR and IFNγ pathway. The glioma cells with detectable CD274 (**D**) or PDCD1LG2 (**E**) showed an elevated activity of “PI3K_AKT_MTOR_SIGNALING”. (**F**) The PI3K_AKT_MTOR_SIGNALING pathway activity was significantly increased in tumor-associated macrophages, instead of T cells.

Immune checkpoints involved in the PD-1 signaling pathway were found to be strong predictive markers for anti-PD-1 treatment and were significantly upregulated in the altered RTK/Ras/PI3K/AKT pathway subgroup (Student’s *t* test, Figure 3A). However, the single cell RNA sequencing results showed that *CD274* and *PDCD1LG2* were not only expressed in malignant cells, but were also heavily expressed in immune cells in gliomas (Supplementary Figure 1A, 1B). So, we analysed the immune checkpoints according to cell type. As GSEA analysis indicated a prominent enrichment of transcript signatures involved in the PI3K-AKT-mTOR and IFN-γ pathways for the TCGA bulk sequencing (Figure 3B), we quantified the signaling pathway of each single cell by ssGSEA [18, 19] using the “INTERFERON_GAMMA_RESPONSE” and “PI3K_AKT_MTOR_SIGNALING” gene sets. There was a significant positive correlation between “PI3K_AKT_MTOR_SIGNALING” and “INTERFERON_GAMMA_RESPONSE” in the malignant cells (Figure 3C). Moreover, malignant cells with detectable *CD274* expression showed elevated levels of “PI3K_AKT_MTOR_SIGNALING” (Figure 3D). However, the activity of this pathway was increased in glioma cells with detectable *PDCD1LG2* expression in the study by Neftel et al [7] but not in that by Filbin et al [20] (Figure 3E). PD-L2 expression was also increased in gliomas with RTK activation in the study by Filbin et al., but the increase was not significant. It is possible that RTK was activated in fewer cells in the study by Filbin et al. compared to that in the study by Neftel et al. Finally, “PI3K_AKT_MTOR_SIGNALING” was significantly increased in tumor-associated macrophages with detectable *PDCD1* but not in T cells (Figure 3F).

### RTK/Ras/PI3K/AKT signaling predicts the immune phenotype and cell survival in diffuse gliomas

**Figure 4:**
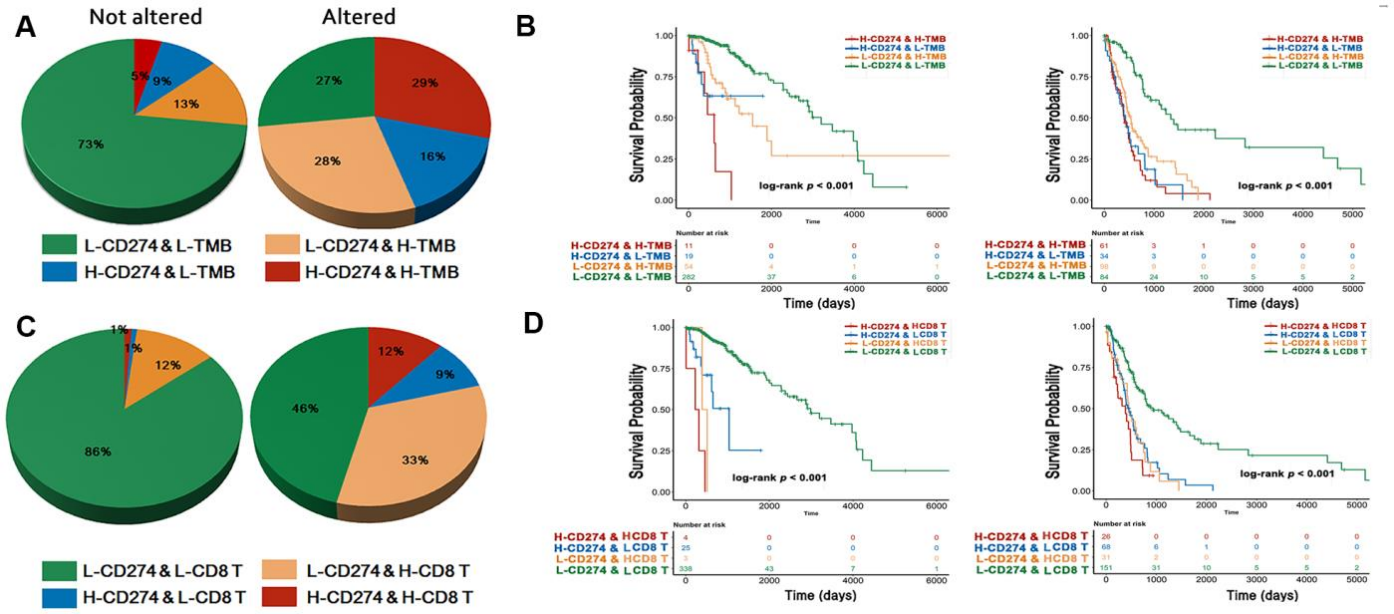
**Immune phenotype and survival analysis.** The immuno-phenotype showed higher CD274 and TMB (**A**) in the tumor with altered RTK/Ras/PI3K/AKT signaling with different survival outcome (**B**). CD274 and CD8+ T cells (**C**) were significantly higher in activated RTK/Ras/PI3K/AKT pathway, with difference in OS (**D**).

CD274, TMB, and CD8^+^ T cell infiltration were selected as markers for identifying the immune phenotype [21–23]. The cut-offs of these predictive biomarkers were determined based on survival outcomes. We observed that the activated RTK/Ras/PI3K/AKT signaling pathway predicted a worse OS for diffuse gliomas (Supplementary Figure 2). Next, we investigated the prognostic value of the immune phenotype according to the status of the RTK/Ras/PI3K/AKT signaling pathway. First, we classified the immune phenotypes based on *CD274* and TMB, which served as a strong predictor for patients with cancers treated with ICIs [21]. Here, we saw that a higher proportion of high-*CD274* and high-TMB samples exhibited alterations in the RTK/Ras/PI3K/AKT signaling group than their counterparts (*p* < 0.001, Figure 4A). However, we found that the group with both high *CD274* and TMB suffered the worst OS compared with other groups, regardless of the RTK/Ras/PI3K/AKT signaling status (*p* < 0.001, Figure 4B). Furthermore, *CD274* and CD8^+^ T cells were used for classifying TME according to previous reports [22, 23]. There was a lower proportion of the group with lower *CD274* and CD8^+^ T cells in tumors without RTK/Ras/PI3K/AKT signaling alterations (*p* < 0.001, Figure 4C). The immune phenotype based on the presence of *CD274* and CD8^+^ T cells also showed different survival outcomes based on whether RTK/Ras/PI3K/AKT signaling was altered (*p* < 0.001, Figure 4D).

### Associations of RTK/Ras/PI3K/AKT signaling with clinical characteristics

**Figure 5:**
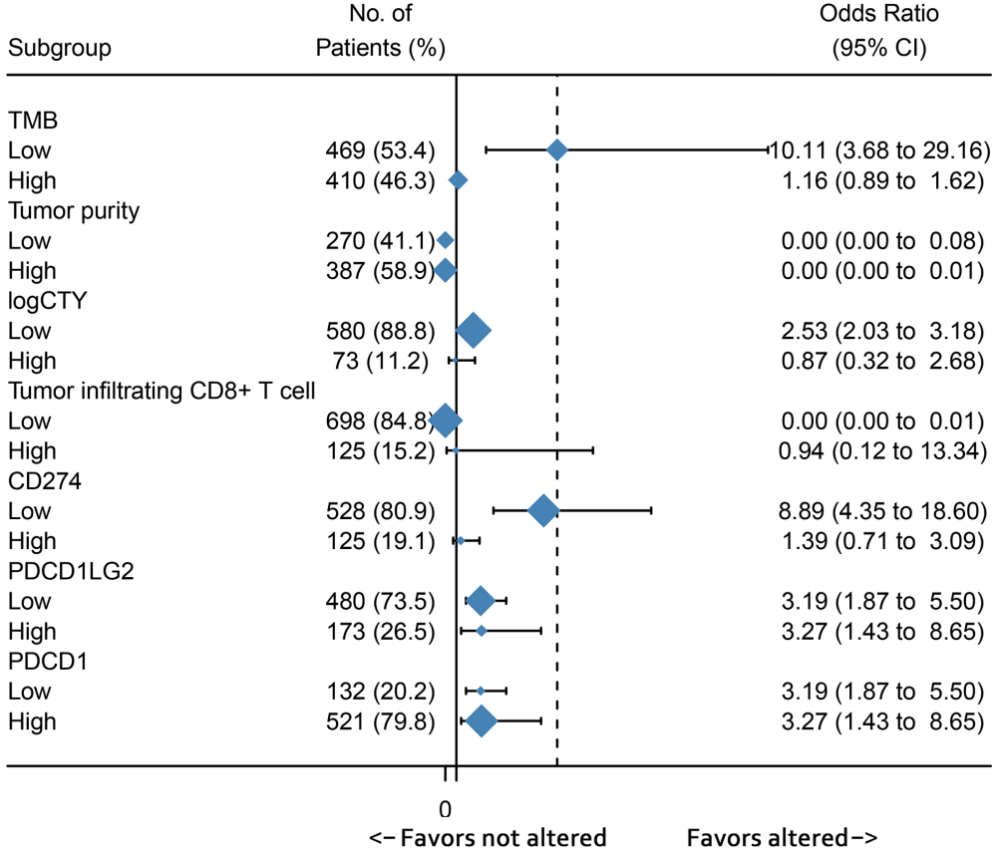
Subgroup analysis showed the association between immunotherapy predictive biomarkers and RTK/Ras/PI3K/AKT pathway.

The predictive immunotherapy biomarkers were divided into two groups according to survival outcomes, and the cut-off values are presented in Supplementary Table 4. We observed that RTK/Ras/PI3K/AKT signaling occurred more frequently in the subgroup with lower TMB, CYT, and *CD274* expression. However, alterations in this pathway were consistently high regardless of changes in tumor purity, and *PDCD1LG2* and *PDCD1* expression (Figure 5).

The association between the RTK/Ras/PI3K/AKT signaling status and the clinicopathological factors of patients is presented in Table 1. Patients with altered RTK/Ras/PI3K/AKT signaling were significantly younger. Moreover, alteration in this pathway was more likely to occur in the neural and proneural TCGA transcript subtype, and in LGG. Additionally, isocitrate dehydrogenase mutations and ATRX chromatin remodeller loss were significantly correlated with activated RTK/Ras/PI3K/AKT signaling (Table 1). Analysis of the prognostic value of the clinicopathological factors by the log-rank test, showed that age, grade, isocitrate dehydrogenase, and RTK/Ras/PI3K/AKT pathways were independent variables for overall survival (Table 2). A nomogram based on these four independent prognostic factors was built, and the C-index for this model was 0.81. The nomogram model effectively predicted the 1-, 3-, and 5-year overall survival rates (Supplementary Figure 3).

**Table 1:** Association of RTK/Ras/PI3K/AKT signaling and clinicopathological factors.

**Variables**	**No (%)**	**RTK/Ras/PI3K/AKT signaling**		
		**not altered**	**altered**	***p* value**
**Age**				
	885 (100%)	52.66 ± 16.11	49.47 ± 15.57	0.003
**Gender**				
Female	363 (41.2%)	152	211	0.581
Male	518 (58.7%)	227	291	
**Grade**				
LGG	507 (57.3%)	201	306	0.020
GBM	378 (42.7%)	180	198	
**TCGA subtype**				
Classical	129 (18.9%)	64	65	0.003
Mesenchymal	145 (21.2%)	72	73	
Neural	133 (19.4%)	46	87	
Proneural	277 (40.5%)	101	176	
**IDH-1 R132H**				
Mutant	439 (50.2%)	157	282	< 0.001
Wild-type	436 (49.8%)	218	218	
**ATRX**				
Loss	206 (25.8%)	69	137	0.005
Expression	594 (74.3%)	265	329	
**MGMT**				
Methylated	563 (68.2%)	225	338	0.059
Unmethylated	263 (31.8%)	124	139	

**Table 2:** Univariate and multivariate survival analysis.

**Variables**	**Univariate analysis**			**Multivariate analysis**	
	**HR (95%CI)**	**p-val**		**HR (95%CI)**	**p-val**
Age > 65	2.09 (1.52 ~ 2.86)	< 0.001		2.09 (1.52 ~ 2.86)	< 0.001
Grade (GBM)	7.45 (5.95 ~ 9.32)	< 0.001		2.26 (1.55 ~ 3.29)	< 0.001
IDH (WILD-TYPE)	9.32 (7.27 ~ 11.96)	< 0.001		3.76 (2.26 ~ 6.25)	< 0.001
ATRX (WILD-TYPE)	3.15 (2.34 ~ 4.26)	< 0.001		0.98 (0.64 ~ 1.48)	0.907
MGMT (Methylated)	2.96 (2.39 ~ 3.65)	< 0.001		1.27 (0.95 ~ 1.70)	0.113
TCGA subtype (PN)	0.58 (0.53 ~ 0.63)	< 0.001		0.97 (0.83 ~ 1.12)	0.633
RTK/Ras/PI3K/AKT signaling	4.82 (3.79 ~ 6.13)	< 0.001		1.56 (1.08 ~ 2.24)	0.017

## DISCUSSION

Pathway-level activities have proven to be more stable than single-gene activities for stratifying patients into subgroups for predicting survival and guiding special therapeutics [24, 25]. In our study, we defined the activation of a pathway by CNV or genetic mutation and excluded unknown significant genetic alterations. Moreover, we performed a GSEA analysis using transcriptomic data to confirm that the two subgroups showed different PI3K_AKT_MTOR_SIGNALING activities. Next, we showed that tumors with altered RTK/Ras/PI3K/AKT signaling are characterized by favourable predictive immunotherapy biomarkers, including TMB, tumor-infiltrating CD8^+^ T cells, immune checkpoints, and interferon-γ signaling. These results indicated a selection of patients for a combination of immunotherapy and treatment with PI3K inhibitors.

Three distinct immune patterns have been previously proposed: immune-inflamed, immune-excluded, and immune-desert [26]. Immune-inflamed tumors are characterized by increased TMB, checkpoint expression, interferon-γ signaling, and CD8^+^ T cell infiltration and are responsive to immunotherapy [26, 27]. Here, we found that gliomas with altered RTK/Ras/PI3K/AKT signaling mainly participate in the immune and inflammatory responses and are classified as immune-inflamed tumors. Moreover, this subgroup of gliomas also exhibited the same patterns as the immune-inflamed tumors in terms of having higher TMB, immune checkpoints, CD8^+^ T cell infiltration, and interferon-γ signaling. The single-cell RNA seq data revealed a positive association between the activated RTK signaling pathway and PD-1 expression in tumor-associated macrophages, and PD-L1 expression in tumor cells. These results indicate that gliomas with active RTK/Ras/PI3K/AKT signaling are responsive to ICI immunotherapy via the targeting of PD-1/PD-L1 both on tumor cells and tumor-associated macrophages. Interestingly, increased RTK/Ras/PI3K/AKT pathway-related gene expression not only increased *CD274* expression in malignant cells, but also increased the expression of *PDCD1* in macrophages in our study. *PDCD1* was shown to inhibit phagocytosis by tumor-associated macrophages, thus providing anti-tumor immunity [28]. Consequently, inhibitors targeting the RTK/Ras/PI3K/AKT pathway in gliomas may exert benefits by activating tumor-associated macrophages.

Numerous inhibitors targeting the RTK/Ras/PI3K/AKT pathway have been developed and tested in clinical trials, but few have achieved a satisfactory therapeutic effect [29, 30]. Immunotherapy based on ICIs for gliomas has also not significantly improved survival in gliomas [31]. However, our results indicated that a combination of these two therapies could exert a better therapeutic effect. Moreover, experimental results showed that combinatorial therapy with nivolumab and inhibitors of tyrosine kinase effectively prolonged the survival of mice with GBM [32]. As nivolumab showed limited effects in patients with GBMs refractory to bevacizumab therapy [31], we suggest that this combination therapy may be applied to patients with altered RTK/Ras/PI3K/AKT pathway expression, who had predictive biomarkers for both treatments.

## CONCLUSIONS

The RTK/Ras/PI3K/AKT pathway is frequently altered in gliomas, which has important prognostic and predictive value for immunotherapy. Moreover, our results indicate that a combination of immunotherapy and treatment with RTK/Ras/PI3K/AKT pathway inhibitors may benefit the survival of patients with gliomas. Clinical studies are necessary to validate the analytical accuracy of our study and examine the clinical utility of our findings in the personalized treatment of gliomas.

## MATERIALS AND METHODS

### Patients and data

The alterations in oncogenic signaling pathways in diffuse gliomas were identified based on copy number variation (CNV) and somatic mutation through the cBioPortal (http://www.cbioportal.org) [33, 34]. The CNV and mutations in genes of unknown significance and germline mutations were excluded when identifying alterations in pathways. The Cancer Genome Atlas (TCGA) PanCancer Atlas database was used, which consists of 661 cases with both mutation and CNV data. One or more gene alterations in the oncosignaling pathways were identified as an “altered pathway” patient sample. The corresponding transcriptome and whole exome sequencing data were downloaded from the GDC Data Portal of TCGA (https://portal.gdc.cancer.gov/). We used the somatic mutation as determined using TCGA as the mutation count per sample. Thirty-five megabases (Mb) was considered as the total exome size. We counted the TMB as the number of mutations/35 [35]. SingleCellPortal (https://portals.broadinstitute.org/single_cell/) was used to download single cell mRNA-seq data from the Neftel et al. study [7] and the Filbin et al. study [20]. Moreover, tumor-infiltrating immune cells were accessed from the Timer website (https://cistrome.shinyapps.io/timer/) [36]. ESTIMATE was used to calculate tumor purity [37].

### Bioinformatic analysis

The package “edgeR” was employed to identify differentially expressed genes with a log fold change > 1 and FDR < 0.05. Gene ontology (GO) was used for the analysis of differentially expressed genes (DEGs) through the DAVID website (http://david.ncifcrf.gov/) [38]. Data were log-transformed before drawing heat maps. Gene set enrichment analysis (GSEA) was used to investigate the potential association between the RTK/Ras/PI3K/AKT pathway status and the immune response in the “hallmark gene sets (h.all.v7.0.symbols)” using Java 4.0 Desktop Application (http://software.broadinstitute.org/gsea/index.jsp) [39]. The threshold for GSEA results was set at NES > 1.5 and FDR < 0.25. GSVA was used to evaluate the pathways in samples with mRNA data using the single-sample gene set enrichment analysis (ssGSEA) method. CYT was calculated by the genomic means of *GZMA* (granzyme A) and *PRF1* (perforin 1) in TPM values [40].

### Statistical analysis

The R (3.5.2) language was used as the main tool for analysing data and drawing figures. The discrepancies in TMB, tumor purity, CYT, and tumor-infiltrating CD8^+^ T cells were compared by the Wilcoxon test using the wilcox.test package. Immune checkpoint expression data were log transformed and compared by Student’s *t*-test. Correlations between CD274/2 and genes involved in the RTK/Ras/PI3K/AKT pathway were analysed by Spearman correlation and correlogram analysis. The cut-off for continuous variables were determined by survival data using X-tile 3.6.1 [41]. Overall survival (OS) analysis was performed using the log-rank test. A *p* < 0.05 was considered as statistically significant.

## Supplementary Material

Supplementary Figures

Supplementary Table 1

Supplementary Table 2

Supplementary Table 3

Supplementary Table 4
