# Supplementary material for: Alterations in the RTK/Ras/PI3K/AKT pathway serve as potential biomarkers for immunotherapy outcome of diffuse gliomas
Source: Aging (Albany NY). 2021 Jun 8;13(11):15444–58. doi: 10.18632/aging.203102 (PMC8221357; doi:10.18632/aging.203102)
Supplement: Supplementary Figures [file aging-13-203102-s001.pdf]

SUPPLEMENTARY FIGURES

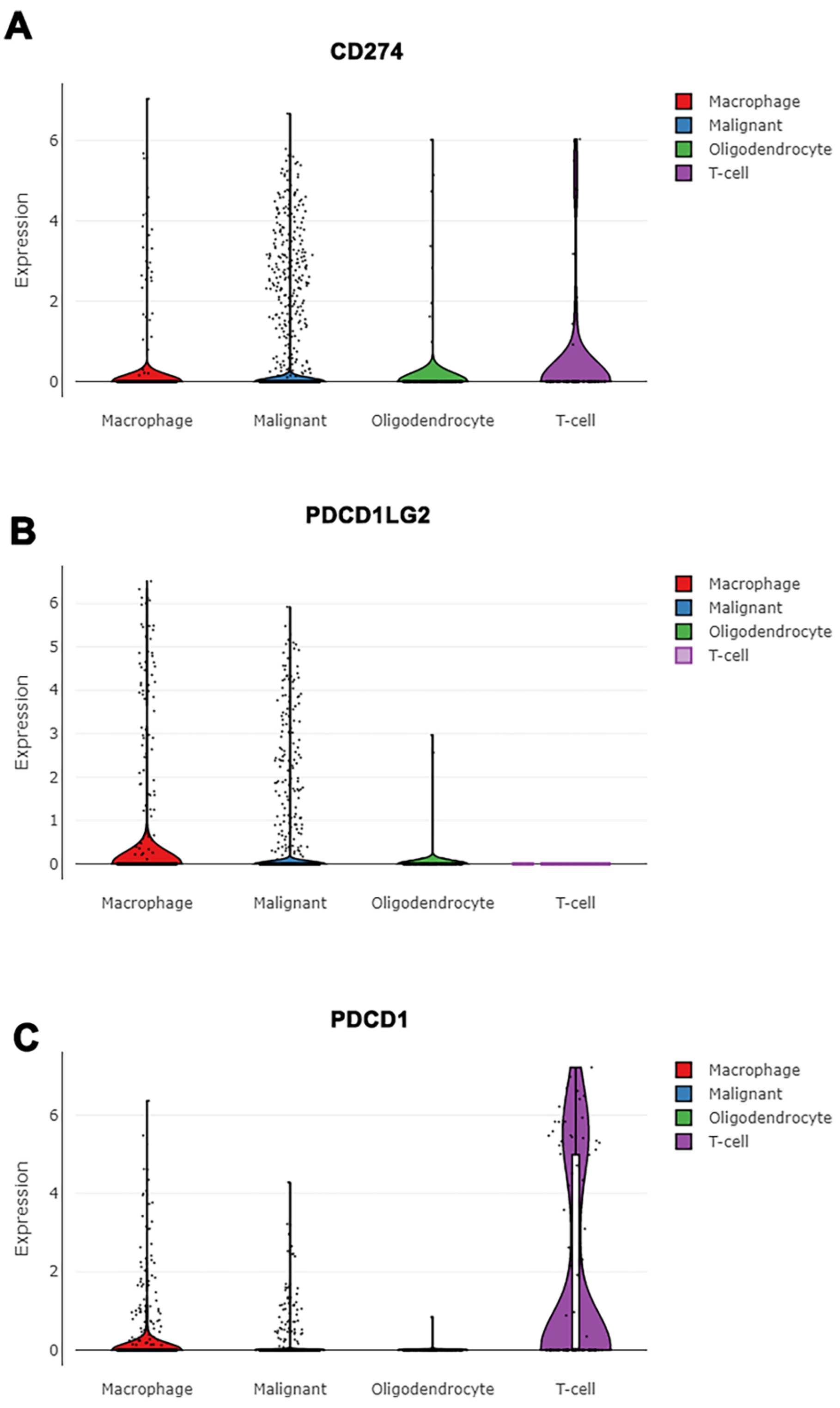

Supplementary Figure 1. The single cell analysis showed the distribution of CD274, PDCD1LG2 and PDCD1 according to cell type. (**A**, **B**) CD274 and PDDCD1LG2 were highly expressed in both malignant cells and immune cells in gliomas. (**C**) The expression of PDCD1 was significantly increased in T cells.

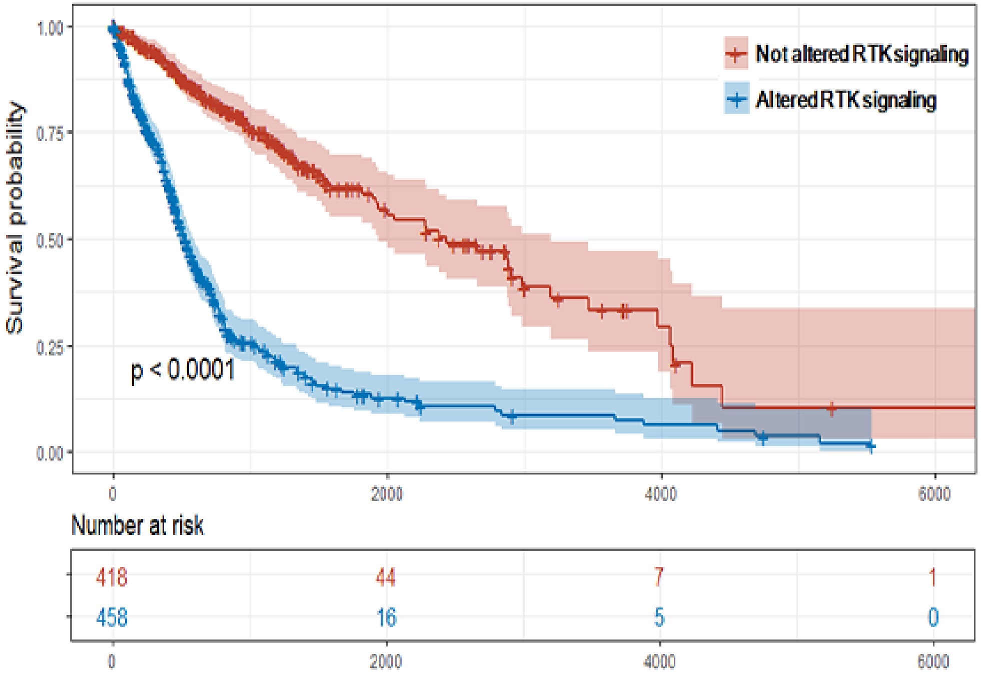

Supplementary Figure 2. The survival analysis of RTK/Ras/PI3K/AKT signaling in gliomas.

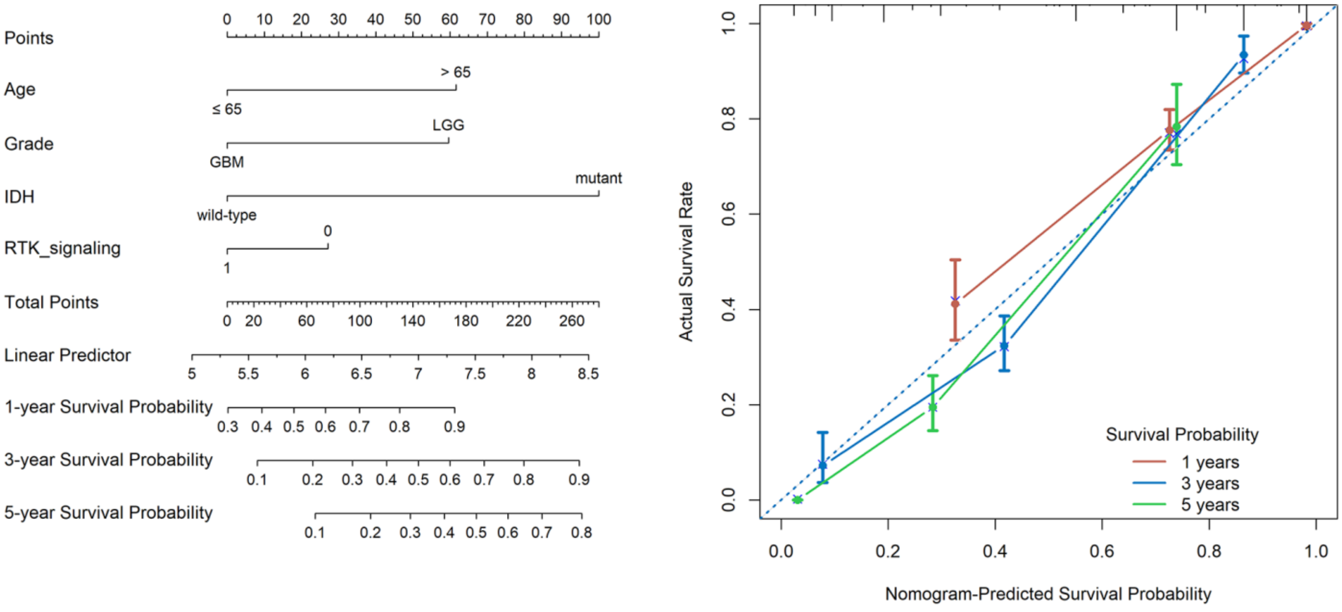

Supplementary Figure 3. Prognostic nomogram for OS and calibration curve for predicting OS.
