# Supplementary material for: Alterations in the RTK/Ras/PI3K/AKT pathway serve as potential biomarkers for immunotherapy outcome of diffuse gliomas
Source: Aging (Albany NY). 2021 Jun 8;13(11):15444–58. doi: 10.18632/aging.203102 (PMC8221357; doi:10.18632/aging.203102)
Supplement: Supplementary Table 1 [file aging-13-203102-s002.pdf]

SUPPLEMENTARY TABLE

Supplementary Table 1. Genes involved in oncogenic signaling pathways.

Oncogenic signaling pathways	Genes
RTK/Ras/PI3K/AKT Signaling	GFR ERBB2 PDGFRA MET KRAS NRAS HRAS NF1 SPRY2 FOXO1 FOXO3 AKT1 AKT2 AKT3 PIK3R1 PIK3CA PTEN
RB pathway	CDKN2A CDKN2B CDKN2C CDK4 CDK6 CCND2 RB1
TP53 pathway	CDKN2A MDM2 MDM4 TP53
