# Supplementary material for: Alterations in the RTK/Ras/PI3K/AKT pathway serve as potential biomarkers for immunotherapy outcome of diffuse gliomas
Source: Aging (Albany NY). 2021 Jun 8;13(11):15444–58. doi: 10.18632/aging.203102 (PMC8221357; doi:10.18632/aging.203102)
Supplement: Supplementary Table 4 [file aging-13-203102-s005.pdf]

Supplementary Table 4. Cutoff values for predictive immunotherapy biomarkers.

Biomarker	cutoff
TMB	1
Tumor purity	0.9
CD8	0.3
logCTY	3.3
logCD274	1.2
logPDCD1LG2	1.4
logPDCD1	0.4
